# Effect of corn or barley in feedlot diets on steer performance and feeding behavior

**DOI:** 10.1093/tas/txaa099

**Published:** 2020-12-22

**Authors:** Hannah M DelCurto Wyffels, Julia M Dafoe, Cory T Parsons, Darrin L Boss, Timothy DelCurto, Samuel A Wyffels, Megan L Van Emon, Janice G P Bowman

**Affiliations:** 1 Department of Animal and Range Sciences, Montana State University, Bozeman, MT; 2 Northern Agricultural Research Center, Montana State University, Havre, MT

## INTRODUCTION

Montana produces approximately 1.1 million feeder calves annually; however, only 5% are retained in state for backgrounding and/or finishing (USDA-NASS, 2018). Calves raised through a backgrounding program consume more feed, gain faster in the feedlot, and produced heavier carcasses with greater profit, than calves put in the feedlot immediately after weaning (Lewis et al., 1990a, b). A variety of feedstuffs can be used in cattle backgrounding/feedlot rations. Corn is the most common feed grain in the United States; however, barley is more widely grown in the northwest, as it is more adapted to the growing and climatic conditions. Montana is the second largest barley producer in the United States (USDA-NASS, 2018), suggesting there is a strong potential for Montana cattle producers to use barley produced in the state to grow calves to heavier weights before selling them or to use barley in finishing diets in local feedlots.

The use of barley, however, has been criticized due to its rapid digestion in the rumen, which can result in digestive disorders such as decreased ruminal pH and lactic acidosis (Kellems and Church, 2003). The extent to which feeding behavior and rate of digestion influences animal performance remains to be elucidated. Previous work has demonstrated that barley and corn have comparative nutritive value and yield similar animal performance [average daily gain (ADG); quality grade; and yield grade; Nichols, 1988; Dion and Seoane, 1992], whereas others have found steers to have poorer performance when fed barley-based diets (Boss et al., 1994). Research at Montana State University has indicated barley has equal or higher energy values than corn in feedlot diets (Boss and Bowman, 1996; Milner et al., 1996; Kincheloe et al., 2002).

Although differences in cattle performance have been observed when using corn and barley rations for finishing cattle, our understanding of the causes of variation in steer performance is limited. In addition, further work is needed to evaluate the extent to which feeding behavior influences animal performance. Golden et al. (2008) found inefficient steers to have greater within-day variation in intake compared with efficient steers. In contrast, Parsons et al. (2004) reported that cattle could not be classified into performance and carcass outcome groups based solely on feeding behavior. Therefore, the objective of this study was to evaluate the effects of finishing diets based on corn or barley on feedlot performance and feed intake behavior. We hypothesized that barley-fed steers would have comparable performance characteristics but perhaps differ in respect to feeding behavior to steers fed a corn-based ration.

## MATERIALS AND METHODS

Experimental procedures described herein were approved by the Agriculture Animal Care and Use Committee of Montana State University (#2016-AA26). All animals used in this study were provided by the Montana Agricultural Experiment Stations, and the study was conducted at the Northern Agricultural Research Center in Havre, Montana.

Angus-based steer calves from two consecutive years averaging 427.3 ± 3.7 kg (*n* = 48) in year 1 and 406.8 ± 3.4 kg (*n* = 47) in year 2 were used in the feedlot trial. Steers from years 1 and 2 were subjected to the same experimental protocol. Upon entry to the feedlot, steers were stratified by weight and assigned to one of two dietary treatments and one of 12 pens. The two dietary treatments included primary basal grains: 1) corn or 2) Hockett barley. Grains were dry-rolled, and diets were formulated to be isonitrogenous (12% crude protein) and contain 80% grain, 12% barley straw, 3% canola oil, and 5% supplement with the exception of year 2 barley ration that contained 10% supplement due to differences in initial protein content. Supplements consisted of vitamin/mineral packages for feedlot steers and protein sources such as wheat middlings and canola meal were added to the supplement to make diets isonitrogenous. Ingredient and nutrient composition of the diets are presented in Table 1. Steers were on trial between 27 February 2017 and 12 June 2017 (105 d) in year 1 and between 26 February 2018 and 11 June 2018 (105 d) in year 2.

**Table 1.** Composition and nutrient content of finishing diets containing corn or Hockett barley as basal grains

	Year 1		Year 2	
	Barley	Corn	Barley	Corn
Ingredient				
Corn	—	80.00	—	75.00
Barley	80.00	—	80.00	—
Barley straw	12.00	12.00	12.00	12.00
Canola oil	3.00	3.00	3.00	3.00
Supplement	5.00	5.00	5.00	10.00
Chemical composition, %				
DM	93.37	93.54	95.41	94.9
CP	10.24	12.30	9.58	12.47

DM = dry matter.

CP = crude protein.

Steers were fitted with an electronic identification ear tags and were adapted to the GrowSafe system (GrowSafe Systems Ltd., Airdrie, AB, Canada) for 14 d prior to the start of the study. Each of the 12 pens consisted of 2 electronic feed bunks, each equipped with an antenna to detect animal presence. Load cells measured feed disappearance and neck bars allowed for only one animal to enter the feed bunk at a time. Individual animal intake was continuously recorded via wireless transfer to a data-acquisition computer. Steers were fed their respective diets at 0800 daily. Diet samples were taken weekly for each treatment throughout the feeding trial for nutrient analysis. Individual steer was considered the experimental unit.

Initial and final unshrunk weights were obtained on two consecutive days and averaged. Steers were weighed every 28 d during the experiment. Dry matter intake, ADG, and feed efficiency (as indexed by the ratio kg weight gain:kg feed intake) were calculated for each steer. Steers were harvested at a commercial abattoir and carcass measurements were collected. An Onset (Bourne, MA) HOBO U30-NRC Weather Station was placed near the feedlot and programmed to collect air temperature, relative humidity, and wind speed and direction data for the entirety of the feedlot trial.

Carcass and performance measurements were analyzed using generalized linear models including year, treatment, and year × treatment interactions as fixed effects. Feeding behavior was analyzed using generalized linear mixed models including year, treatment, and year × treatment interactions as fixed effects with individual steer as a random intercept to account for autocorrelation of multiple measurements from each individual. Least square means were separated using the Tukey method when *P* < 0.05. All statistical procedures were conducted in R (R Core Team, 2017).

## RESULTS AND DISCUSSION

Average daily gain and final live weight differences displayed year effects (*P* < 0.01; Table 2). This observation was likely due to prolonged weather events experienced during year 2. In year 2, March and April had lower mean and minimum daily temperatures than year 1. Greater precipitation was also present in every month during year 2 compared with year 1 (Table 3). The performance differences noted each year are in agreement with that of Milligan and Christison (1974) who found ADG was correlated with mean ambient temperatures, days below −23 °C, windchill, and dewpoint. Additionally, environmentally prolonged cold stress coupled with moisture has been demonstrated to decrease daily gain by approximately 10% (NRC, 2016).

**Table 2.** Performance and carcass characteristics of steers consuming finishing diets containing corn or Hockett barley as basal grains at the Northern Agricultural Research Center

	Treatment			*P*-value		
	Barley	Corn	SEM	Treat	Year	Treat × year
Performance						
Initial wt, kg				0.67	0.01	0.73
Year 1	425.73	428.80	5.01			
Year 2	406.99	406.54	5.07			
Final wt, kg				0.10	<0.01	0.96
Year 1	611.59	628.13	6.96			
Year 2	581.29	597.04	7.04			
ADG^1^				0.04	<0.01	0.84
Year 1	1.77^a^	1.90^b^	0.04			
Year 2	1.57^a^	1.72^b^	0.04			
G:F	0.14	0.14	0.002	0.88	0.98	0.11
Carcass						
Hot carcass wt, kg				0.01	<0.01	0.820
Year 1	356.56^a^	373.51^b^	4.63			
Year 2	338.03^a^	352.86^b^	4.68			
Marbling				0.07	0.03	0.20
Year 1	427.50	474.17	17.9			
Year 2	483.49	483.75	18.1			
Fat, cm	0.94	1.09	0.06	0.15	0.67	0.87
REA^2^, cm^2^				0.38	<0.01	0.70
Year 1	90.78	88.98	1.43			
Year 2	84.04	83.36	1.45			
Yield grade	2.40^a^	2.74^b^	0.08	0.02	0.18	0.67

^1^ADG = average daily gain.

^2^REA = rib eye area.

^a,b^Means with superscript differ (*P* < 0.05).

**Table 3.** Temperature and precipitation patterns for the finishing period during two consecutive years at the Northern Agricultural Research Center

	February	March	April	May	June
Year 1					
Precipitation, cm	0.68	0.14	0.66	0.34	0.68
Temperature, °C					
Mean	−8.30	2.41	7.52	14.56	18.78
Min	−12.09	−15.47	−4.23	−0.96	5.08
Max	−1.84	24.73	24.53	33.26	37.32
Year 2					
Precipitation, cm	1.61	0.64	0.54	1.31	1.32
Temperature, °C					
Mean	−5.43	−5.00	3.94	16.18	17.85
Min	−12.60	−19.79	−18.88	−0.70	6.28
Max	0.85	7.59	30.14	31.15	34.28

Corn-fed steers displayed greater ADG (*P* = 0.04; 1.81 ± 0.03 vs. 1.67 ± 0.03 kg∙d^−1^) and average daily DMI (*P* = 0.02; 11.53 ± 0.16 vs. 11.11 ± 0.16 kg∙d^−1^) than barley-fed steers. No treatment effect was observed for time spent eating, visits per day, time per visit, eating rate, or intake per visit (*P* ≥ 0.08; Table 4). However, significant differences were observed between study years for eating rate and intake per day (*P* ≤ 0.01; Table 4). Visits per day and time per visit displayed year by treatment interactions (*P* ≤ 0.05; Table 4), where barley steers visited the feeder more often and spent less time per visit than corn-fed steers in year 1, with no effects observed in year 2 (*P* > 0.10; Table 4). Limited work has been published relative to feeding behavior for cattle fed barley-based diets. Schwartzkopf-Genswein et al. (2011) evaluated eating behavior of steers fed barley-based backgrounding and finishing diets and found steers with more variable eating patterns exhibited greater ADG and tended to have greater G:F. Although this finding is contrary to industry perception, it may yield potential in sorting calves into performance groups based on eating behavior. Additional work identifying the relationships between feeding behavior and performance of individual animals will be needed to further clarify the impact of feeding behavior on cattle performance.

**Table 4.** Feeding behavior of steers consuming finishing diets containing corn or Hockett barley as basal grains at the Northern Agricultural Research Center

	Treatment			*P*-value		
	Barley	Corn	SEM	Treat	Year	Treat × year
Time spent eating, min/d	103.65	106.07	2.45	0.51	0.34	0.82
Visits per day				0.16	<0.01	<0.01
Year 1	17.27^a^	15.63^b^	0.36			
Year 2	14.88^a^	15.53^a^				
Time per visit, min				0.15	<0.01	0.05
Year 1	3.47^a^	3.80^a^	0.17			
Year 2	4.47^a^	4.08^a^	0.20			
Eating rate, g/min				0.72	<0.01	0.26
Year 1	117.07	114.64	4.90			
Year 2	88.59	95.46	3.90			
Intake per day, kg				0.02	<0.01	0.18
Year 1	11.53^a^	12.24^b^	0.22			
Year 2	10.69^a^	10.81^a^	0.22			
Intake per visit, g	400.71	412.02	8.39	0.08	0.54	0.14

^a,b^Means with superscript differ (*P* < 0.05).

For our study, year differences were observed for hot carcass weight, marbling, and rib eye area (REA; *P* ≤ 0.03; Table 2) but did not affect fat deposition or yield grade (*P* ≥ 0.18). No differences among diets were detected for fat thickness, marbling, or REA (*P* ≥ 0.07). Carcass weights were on average 15.89 kg heavier for steers fed corn compared to steers fed barley (*P* = 0.02), and there was a difference in yield grade with corn-fed steers having a higher numerical yield grade (*P* = 0.02; Table 2). Boss and Bowman (1996) reported similar differences in carcass weights between corn- and barley-fed steers; however, other studies have reported no differences in carcass characteristics due to grain source (Ovenell-Roy et al., 1998; Surber et al., 1998). These performance findings are similar to a meta-analysis conducted by Bowman et al. (unpublished data), that found, when summarizing numerous feedlot trial data, corn-fed steers had a consistent pattern of higher ADG, G:F, HCW, KPH, 12th-rib fat thickness, and yield grade. Presumably, this may be due to increased intake by corn-fed cattle.

## IMPLICATIONS

Steers on corn diets had higher ADG, HCW, and carcass quality grades, but consumed more feed than steers on barley-based diets. As a result, feed efficiency was similar for barley- versus corn-based diets. Thus, depending on cost and production year, barley could be a potential high-quality feed source in beef cattle finishing rations. Year can have a significant impact on both performance and carcass measurements, presumably due to environmental differences across years. Further analysis will be conducted to evaluate the impact weather and environmental conditions have on animal feed intake behavior, rumen function, and performance.
